# Results of Experiments on Pyæmia

**Published:** 1872-07

**Authors:** 


*Results of Experiments on Pyœmia.* Pathological Society of London, Tuesday, May 7th, 1872.

Dr. Burdon Sanderson delivered an address on this subject relating his first experiments as to the effect of inoculating animals with pyæmic liquids. In the autumn of 1867, he injected the purulent liquid contained in the ankle-joint of a patient, who had died a few hours before with metastatic abscesses, general suppurative arthritis, and intense septicæmia, under the skin in a dog and two guinea-pigs. The two guinea-pigs died within fifteen and twenty days. Both had metastatic abscesses; in one the lungs were beset with minute nodules, resembling miliary tubercles. The dog lived seven weeks; there were no secondary abscesses, but miliary tubercles of the liver and spleen. From one of the guinea-pigs, two others were inoculated; one died of pyæmic subcutaneous abscesses, without visceral disease; the other lived longer, had no abscesses, but tuberculous disease of the lungs. During the same winter, other experiments were made, which seemed to show that, by the inoculation of pyæmic products two sets of lesions might be produced: as an immediate result, metastatic abscesses, accompanied by a general typhoid state, which was often fatal; as an ulterior result, either disseminated nodules, at first hard, but afterwards becoming caseous at their centres, or interstitial induration—both forms of lesion having their seat chiefly in the lungs, spleen, and liver, but also occurring in other viscera.

Dr. Sanderson next said that experiments were made in 1871, as to the existence of bacteria in animal liquids, and the circumstances had shown that, whereas bacteria could not be proved to be present, either actually or in germ, in the healthy liquids or tissues, or in the products of healthy inflammation, they were present potentially in pyæmic liquids—that is, whereas ordinary pus could be kept for days or even weeks free from bacteria, provided the precautions against "spontaneous generation" were observed, pyæmic pus could not be so kept, and, moreover, possessed the properties of at once determining the development of bacteria in any suitable liquid to which it was added.

On the last occasion on which he had brought the subject of the intimate pathology of tubercle before the Society, he argued that tuberculosis is an irritative overgrowth of a pre-existing tissue. It had then been shown that the process, in its disseminated or interstitial form, had its seat in a certain tissue, and this tissue had been termed adenoid or lymphatic—both words implying its intimate and special relation with the lymphatic system; but the precise anatomical nature of this relation had been imperfectly made out. Last May Dr. Klein came to England with the object of co-operating in the investigation of this very question. The field taken up was the peritoneum; the reason of the choice being, that that membrane, and especially the omentum and diaphragm, had already been the subject of investigation, as favorite seats of tuberculosis. Those researches had not merely served to elucidate one or two anatomical facts of very great importance to the pathologist—*e. g.*, the existence of a lymphatic system in the omentum and its distribution, and the mode in which the peritoneum communicates with the lymphatic system—but had rendered it possible to give an account which, so far as the peritoneum was concerned, was tolerably exact and complete, both of the normal process of absorption and of the changes which the absorbing tissues undergo when they are entered by infective agents.

It was found that in one set of cases, the secondary lesions were suppurative, the constitutional disturbance intense, and the fatal result rapid; in another, that the lesions were vascular new growths, firm at first, afterwards becoming caseous, the progress slow, and the functional disturbance imperceptible. And then it appeared that in all those instances in which the pyæmia—*i. e.*, the acute character—manifested itself, bacteria were present, not merely in the purulent liquids, but in the blood. Under these circumstances, attention was directed from effects to the poison itself. Soon after the opening of the Brown Institution, it was found that the practice of the hospital for animals was likely to afford the required material; in short, that pyæmia occurred in dogs under circumstances very similar to those which determined it in human beings; and exhibited similar symptomatical and pathological aspects. A series of experiments were therefore commenced in January last, having for their object to acquire a knowledge of the morbid poison, and particularly to discover by what conditions the variations of its intensity were governed.

Dr. Sanderson proceeded to say: "The word pyæmia is apt to be used in somewhat different senses, according as the person using it has before him the medical or surgical aspect of the disease. To define it completely we must, I think, take into account its mode of origin, its symptoms, and the anatomical changes which it produces; not confining our attention to either of these to the exclusion of the rest. With this consideration in view I would comprehend in my definition the following propositions:—

"1. Pyæmia originates by the introduction into the living tissues, and eventually into the blood, of a *poison* which is itself a *product of inflammation*.

"2. The action of this poison manifests itself in an alteration of the blood, and in disorder of the vital functions. The former of these is characterized by the presence of bacteria, and by change in the optical characters of the blood, which often becomes obviously more transparent and darker by reflected light than it is naturally. Of the latter, viz., the general disorder of the vital functions, the most prominent phenomenon is fever, which, in the more intense forms of the affection, is followed by collapse which culminates in death.

"3. More remotely, the disease manifests itself in secondary suppurations, *i. e.*, in the formation of metastatic abscesses, which may occur either in the internal organs or underneath the skin. The special characters of these metastatic (as I am in the habit of calling them) infective abscesses, are those which are well known both to surgeons and physicians. They have the additional less known character, that the pus they contain is full of bacteria.

"Pyæmia *differs* from tuberculosis in the rapidity of its progress, and in the obvious character of the anatomical changes of which it consists. Whereas by tuberculosis we are understood to mean anatomically the overgrowth of cells in certain tissues, which we designate lymphatic on account of their proved anatomical relation to the lymphatic system, the secondary inflammations of pyæmia result in the formation of infective abscesses.

"Pyæmia resembles tuberculosis in its mode of origin. Both spring from inflammations; and, so far as relates to the anatomical characters of the lesions, both are inflammations. To both, therefore, the term secondary or infective inflammation is applicable.

"So much for the disease itself. Let me now," said Dr. Sanderson, "draw your attention to the nature of the poison. I wish to show (1) that every pyæmic abscess contains a poison, to be present in the products of acute secondary inflammation in the lower animals, also exist in similar products in man? The second is more important still—Can it be shown that human pyæmic products, when tested by inoculation, possess exactly the same morbific properties as those which are possessed by the liquids to which our experiments relate? It is for answers to these inquiries that I earnestly ask the assistance of hospital surgeons.

"This intensification is effected by a process which may be called cultivation. Dr. Klein made the important discovery that, if a pyæmic liquid were transferred to the peritoneum of a guinea-pig, and allowed to remain there for a couple of days, although it did not at first produce any intense symptoms in the animal itself, its toxic intensity increased in such a degree that, when the transudation-liquid produced in this was injected into another animal, it had acquired the most deadly activity; and that all such extremely active liquids were crowded with bacteria of a particular character, the increased number of which seemed to be in proportion to their toxic properties."

Dr. Sanderson then exhibited a dog, into the abdominal cavity of which six drops of a pyæmic transudation-liquid had been injected three hours before. The animal was in a state of profound collapse, accompanied with vomiting, purging, and cramps of the extremities. Shortly afterwards, the animal was killed and the abdominal cavity opened. The peritoneum contained liquid slightly stained with blood, which, on microscopical examination, was found to be crowded with bacteria. The intestines were distended with a frothy liquid, which possessed none of the characters of the natural contents which had been found in other cases to be charged with shed epithelium. The internal surface of the whole of the alimentary canal from the stomach downwards was intensely injected, and presented appearances which (as had been found by more careful investigation in previous cases) were due to the separation of the epithelium from the surface of the mucous membrane, and the infiltration of that tissue with liquid.

The material which produced these results was obtained as follows: Pus from a pyæmic abscess of spontaneous, *i. e.*, accidental, origin was introduced into the peritoneal cavity of a guinea-pig, and allowed to remain there for two days. It was then withdrawn from the guinea-pig, and some of it at once injected into the peritoneum of a dog. The dog was affected in exactly the same way as the animal exhibited to the Society. The remainder of the liquid was kept for five weeks in hermetically sealed tubes, after which six drops were injected into the peritoneum of a guinea-pig; this showed its action to have become relatively feeble. After two days (the day before the meeting) the transudation-liquid produced was tested with a third guinea-pig and found to be extremely active. On the afternoon of the meeting it was injected into the peritoneum of the dog exhibited.

"Such are the facts," said Dr. Sanderson. "The all-important question remains—Do these experiments concern us as physicians and surgeons or not? I think they do. But what I want is to prove it; for I am well aware that, unless clinical observation come in aid of pathological experiment, the results of the latter do not tell practically. Let me state what are the lines of inquiry which I desire to see taken up. The first question is—Do the characters which we have shown, when introduced either into the circulation or into a serous cavity, produce the symptoms of pyæmia? and (2) have we this poison so entirely in our possession, and so far under control, that, beginning with an agent so mild in its action that it produces no marked symptoms, we can convert it into an agent of such intensity that it kills in two or three hours with the formidable symptoms seen in the case we have now before us?

"Finally, I would say a word as to the limits of the question now before us. With regard particularly to the question of bacteria, I desire to keep to the bare facts of disease, and not to diverge into discussions as to their origin. It is a matter to me of comparative indifference how they originate. Our observations lead us to conclude, first, that they afford a *characteristic* by which we may distinguish the products of infective inflammations from those which are not infective, and that their number affords an indication of the degree of infectiveness; and, secondly, that their presence in the blood is an indication of that constitutional disturbance which accompanies infective inflammation—not merely when that disturbance assumes the degree of intensity of which we have an example before us, but in the slighter form of irritative fever. If these facts prove to be true, not only in the lower animals but in man, their importance is quite unaffected by any theory which we may entertain as to the origin of bacteria."

The President said Dr. Burdon Sanderson had entered upon a large inquiry in a clear and distinct manner. It was a striking fact that five or six drops of this peritoneal fluid should produce upon a dog the effects that had just been seen.

Dr. Crisp said he differed from Dr. Sanderson in several of his conclusions. In the first place he saw little or no resemblance between pyæmia and tuberculosis; they had scarcely one feature in common—the one a highly infectious disease, and the other not communicable. There were some in the profession who thought otherwise, but the statistics relating to the attendants at the Brompton Hospital (Medical and non-Medical) were sufficient to refute this conclusion. Pyæmia, too, was uncertain as to the time of its appearance; it might occur from the second day to the third week after an operation, and after a battle the first thousand wounded might be free from it, and the remainder be more than decimated by it. It should also be known that there was a great difference between tubercle in man and that in the lower animals; he had shown long ago that in monkeys and other animals no bleeding occurred from the lungs, that cavities were comparatively rare, that the liver and spleen, rarely affected with tubercle in man, were frequently so in the lower animals, and that in other particulars there were important differences. He (Dr. Crisp), as shown in their "Transactions," had inoculated guinea-pigs with pure pus from a whitlow on his own finger, and had produced tubercle, but in many respects it differed from tubercle in the human subject, although microscopically it was the same. He had also performed numerous inoculations in birds and in other animals, the results of which led him to the same conclusion. It was also important to bear in mind that there was a wide distinction between diseases of the lower animals and those affecting the human subject. Pyæmia in the lower animals was of rare occurrence, although millions of animals were castrated and spayed yearly. Pigs had the ovaries removed, and salt was often rubbed into the wound, and he (Dr. Crisp) should have more faith in the use of salt during the prevalence of pyæmia than in the use of carbolic acid or in any application hitherto tried. What we wanted were experiments to ascertain whether by the use of chemical agents we could render this poison innocuous—whether by any external application we could prevent the occurrence of pyæmia. Again, as regards *bacteria* being the cause of pyæmia, he entirely differed from Dr. Sanderson. *Bacteria* were found in numerous diseases of the lower animals, as had been fully shown by the French pathologists, and they were probably the effects and not the cause of the disease, as he (Dr. Crisp) had long since endeavored to show. He was the first in this country to describe splenic apoplexy, and to point out its deleterious effects upon man and other animals. Experiments had been made by French pathologists, who showed that after the inoculation of rabbits with the blood of animals dying of this disease, that *bacteria* were found in the blood after a certain time, and that death took place at a given period. Dr. Sanderson's experiments appeared to him to have no important bearing upon pyæmia, the irritating matter he used acted more like a poison, such as prussic acid or arsenic, and killed nearly in a definite time, as others had shown before. To show how differently various substances acted when introduced into the body, he might mention that, unhappily, very recently in his own neighborhood, a gentleman in opening an erysipelatous abscess of the scalp, received some of the pus into his eye, and he was dead in four or five days. He recollected a similar example that occurred to a French surgeon when he (Dr. Crisp) was attending the Paris hospitals. Many years since, two gentlemen at the Zoological Gardens dissected a lion—one died in a short time from blood poisoning, and the other did not recover for four or five years after the reception of the virus. After this, about fifteen years ago, many of the carnivorous animals (lions, tigers, and jaguars) at the Zoological Gardens died after a short time of blood poisoning, which he (Dr. Crisp) believed was occasioned by their eating the flesh of glandered horses. He dissected several of these, and, as there was a doubt about the nature of the disease, he inoculated a cat and two rabbits, which animals died quickly, or rather, were put to death with all the symptoms of glanders.

Dr. Bastian asked Dr. Sanderson whether he saw in the blood of animals suffering from pyæmia real bacteria or only granules. In the present state of science this was a fundamental distinction. Again, what relation did these bacteria bear to pyæmia as a disease? Were they to be regarded as causes of pyæmia or as consequences? Again, granting that bacteria existed in the blood in pyæmia, and that they were its causes, how did the contact of these bacteria cause pyæmic processes? Bacteria were constantly in communication with open wounds and mucous surfaces; they existed abundantly in the mouth and back of the throat of every individual, and if they caused pyæmia nobody would be able to live. Many experiments had been made in which fluids containing actual bacteria had been injected into animals and no disease had been produced. Dr. Bastian had injected several drops of fluid teeming with bacteria under the skin of frogs, and no disease had been produced. No one in this room, he supposed, would say that it was an habitual thing to find bacteria in the blood of persons dying of typhoid fever or rheumatic fever; and yet in both of these diseases where the temperature had been high during life, in a few hours after death bacteria had been found in abundance in the internal organs. How were those bacteria produced in the blood in these cases, granting that they were essential to the production of pyæmia? How was it to be supposed that the infection was brought about? On the other hand, in cases where the health suffered, and where there was an open wound, there was always the likelihood of changes taking place in consequence of poisonous absorption through the wound.

Mr. Hulke asked Dr. Sanderson if he had not confounded pyæmia and septicæmia. It seemed to him that the dog suffered from the latter, and not from pyæmia. If perfectly filtered pus were injected into an animal, the ordinary symptoms of pyæmia were produced, and the animal recovered; but if the pus were unfiltered, these symptoms were produced *plus* others—such as multiple abscesses, constituting septicæmia. Any putrescent fluid, animal or vegetable, would produce the same result.

Mr. Spencer Wells having performed an operation, after he had partially opened the wound and inserted an elastic catheter, removed an ounce of reddish fluid. Dr. Richardson prepared from it an alkaloid, one *milligramme* of which killed a rabbit. It had effusion into its peritoneal cavity, and a little of this killed another rabbit into which it was injected. A third was killed from the fluid from the second; and a fourth from the fluid from the third; but the fifth recovered after the injection of fluid from the fourth rabbit.

Dr. C. J. B. Williams remembered a *post mortem* examination in University College Hospital, after which the operator complained of a tingling and heat in his hands. The fluid in this case was examined and found to be intensely acid, and to contain many bacteria.

Dr. Murchison had often had an opportunity of examining the bodies of patients who had died of pyæmia following typhus fever, in which there were no ulcerated surfaces, no bed-sores, and no open wounds whatever, and yet in these, pus had been deposited in the joints, under the skin, and sometimes in the internal organs. Every one who had observed epidemics of typhus fever must have seen that pyæmia followed many cases, and that when one case had occurred in a hospital there were many.

Mr. Henry Lee thought that Dr. Sanderson's experiments established that the products of inflammation differed from those of the putrid element, this last being much more fatal.

Dr. Anstie had seen all the symptoms of pyæmia produced where there was no wound whatever, and where no putrid fluid could have entered the system. The only assignable cause sometimes was a cold. Other cases come from exposure to drain smells. He mentioned one case at Westminster Hospital brought from a particular club-house, in which the underground premises were exceedingly unhealthy. A series of cases of a similar kind had come from the same house.

Dr. Cayley wished to know what effect the poisonous fluid had on the animal from whose abdomen it had been taken.

Dr. Sanderson in reply said, he was well aware, from his own experiments, that the ordinary bacteria of putrefaction possessed no toxic action, and that liquids containing them could be injected into the circulation of living animals without result. As regarded the bacteria of pyæmic products, he had carefully guarded against the inference that they were the efficient causes of pyæmia. He regarded them only as characteristic inhabitants of infective liquids, and therefore very probably carriers of infection. He understood the word septicæmia to mean a state of the blood which was only present in the most intense forms of pyæmia, and agreed with Mr. Hulke in regarding metastatic abscesses as an accident rather than as an essential of pyæmic infection. The theory that the pyæmic poison was dependent on an alkaloid, would be disproved in case it should appear that it was incapable of diffusion, but further inquiries were necessary.

The following week, May 21st, Dr. Sanderson gave a supplementary statement. His object was to show the presence of bacteria in the infecting fluid of pyæmia, in the blood of living pyæmic patients, and in pyæmic abscesses; and, secondly, to prove that these living organisms were not introduced into the body from without, but were apparently produced within the body. It will be remembered that in certain experiments recorded in one of the reports of the Medical Officer of the Privy Council, Dr. Sanderson had shown that bacteria were introduced into fluids by contact with impure surfaces, and that when glass-rods, test-tubes, and the like, had been heated before being brought into contact with fluids, no bacteria were therein developed. Dr. Sanderson, we believe, had taken care in his experiments on pyæmia to avoid the introduction of germs from without into the peritoneal cavity. He first showed the liquid taken from the peritoneum of a guinea-pig twelve hours after the injection of fluid into it from the peritoneum of a kitten in which peritonitis was excited by the injection of peritoneal fluid. This liquid was swarming with minute bacteria, chiefly spheroidal, this being the feature of the liquids possessing the most intense infective character. The second specimen was the blood of an animal infected as the guinea-pig, and bacteria existed in the blood of the living animal taken from the heart. The existence of these organisms in the blood was regarded as the index of the general state of infection. The third fluid was that contained in the peritoneum of a guinea-pig twelve hours after injection into that part, of the subcutaneous fluid formed in connection with an abscess produced by the introduction of the solution of ammonia beneath the skin, the injection being made with calcined instruments, and with the special object of avoiding the introduction of external agents; and this fluid contained bacteria. A fourth fluid was exhibited—viz., the liquid from an abscess of the thigh of a puerperal woman, removed seven weeks after her confinement, the fluid being obtained through a fine canula into a glass tube, both of which had been superheated, the latter being at once closed. This fluid contained living organisms in it at the first moment of examination.—*The Doctor*, London, June, 1872.

